# Some Further Notes on the Hydrides

**Published:** 1894-06-23

**Authors:** Benjamin Ward Richardson


### Some Further Notes on the Hydrides.

By Sir Benjamin Ward Richardson, M.D., F.R.S.

Some account has been given of the general properties of the hydrides and especially of the hydride of amyl. Regarding this last hydride some further notice should be offered bearing on the local employment of it, because as a local remedy it possesses many valuable properties.

As amyl hydride boils at a temperature from ten to twelve degrees Fahr. lower than the temperature of the human body it becomes an excellent substance for the production of local insensibility by the method of freezing the surface of the body in order to cause local anæthesia. I have employed it for that purpose during many years, and it is so rapid in its action that ethyl chloride in its new application seems to me to possess no superiority over it. As a rule, the direction of the spray of the hydride in a vigorous stream on the skin leads to insensibility in from six to eight seconds, and it is quite possible to keep up the effect even on a divided surface since the fluid is perfectly insoluble, and is incapable of exciting any irritation. The disadvantage of it is that it freezes the skin rather too rapidly so that the deeper tissues do not get affected as they do when a heavier fluid is used; but, as I have already explained, a mixture of the hydride can be made with ethylic ether, which gets over the difficulty.

Another topical service renderable by amyl hydride is to use it as a solvent of various substances that have to be applied to the surface of the body. Spermaceti, camphor, and many bodies of that class dissolve in the hydride with the utmost facility, yielding a solution which on exposure precipitates the dissolved substance in the most even manner. I have often prescribed a solution of this kind for burns, and once after an accident I used it on myself with most agreeable effect. The solution was made as follows: Camphor and spermaceti were dissolved with four ounces of amyl hydride until a slight precipitate was formed, showing that saturation had taken place. Then two fluid ounces of olive oil were added, and when the whole had well comingled pieces of lint were dipped into the solution and were spread over the burned surface. The cold produced by the evaporation of the hydride gave instant relief to the pain, and when the evaporation was complete a fatty covering was left over the injured part that entirely excluded the air and led to recovery without once removing the dressing. A return of the burning sensation was immediately relieved by letting a little of the pure hydride run over the lint and evaporate from it. Care must, of course, be taken in this practice that a light be not brought near the dressing, the solution being readily inflammable; except for this inconvenience all is plain and simple.

There are some other bodies that dissolve in amyl hydride. Iodine is one of these, and the solution formed becomes one of the best for this metalloid. The iodine may be added in the proportion of ten grains by weight, to one fluid ounce of the hydride, and may be applied directly or on cotton wool, to a decomposing wound or open cavity; a fine layer of equally distributed iodine will be left behind. I remember a case in which after the operation of tying a bunch of veins in the scrotum, by an eminent surgeon, there was rather a free venous hæmorrhage a few hours afterwards. The exuded blood coagulated and rapidly decomposed. The surgeon was anxious not to remove the clot and was equally anxious also to avoid the danger of poisoning by absorption. I proposed, thereupon, to pour into the decomposing mass half an ounce of the solution of iodine made with the hydride. We did so, leaving the iodine stranded throughout the whole structure and with the best effect. There was immediate deodorisation, some contraction of the blood clot, and, finally, exfoliation, I had almost said, of the clot with good natural healing when all the clot had been thrown off. The hydride solution of iodine can also be used as spray; the solution need not be saturated with iodine, five grains of iodine to two fluid ounces of the solvent being sufficient. A drachm of such solution can be sprayed carefully into the throat in cases of sloughing or phagedænic ulceration. The iodine is thus made to diffuse itself over all the affected surface to the deepest portion; and left there it is perfect as an antiseptic, destroying putrefactive particles, and causing no recurrence of imflammatory changes.

By bringing strong solution of ammonia in contact with amyl hydride the ammonia is either dissolved or suspended through the solution, so that when the water is separated there is a moderately strong ammoniacal spirit left, which yields a vapour having such powerful antiseptic properties, that diffused through an anatomical jar provided with a good stopper, it will preserve a fresh specimen quite free of change for several days, so free in fact, that even microscopical work can be carried on with it at leisure. In the hurry of practice this is a great advantage. A specimen newly or roughly mounted can be placed under a small bell jar over a cup containing a few drachms of the ammoniacal hydride, and may be returned to after several hours to be found unchanged. A solution of camphor, made by adding camphor to saturation, makes another valuable antiseptic medium especially useful to the naturalist who may dip small specimens into it, assured that when he takes them out of it they are saturated with camphor exquisitely diffused through every part, and are structurally uninjured.

I need make no apology for dwelling at so much length on the value of hydride of amyl. It is a substance so little known in practice, it has so many practical applications—some beyond what I have described—and is now so cheaply obtained, it would, I am sure, be in the hands of all practitioners, and in every hospital if it were properly appreciated. A little attention is required in keeping it owing to its volatility. It should be kept in a well-stoppered bottle having a narrow neck and a small finely ground stopper, and it should be stored in a cool place.

